# Lack of Association between rs4680 Polymorphism in Catechol-O-Methyltransferase Gene and Alcohol Use Disorder: A Meta-Analysis

**DOI:** 10.1155/2020/8850859

**Published:** 2020-11-17

**Authors:** Xin-Rong Jin, Zhi-Qiang Zhao

**Affiliations:** Xinjiang Mental Health Center and Urumqi Fourth People's Hospital, Urumqi 830002, China

## Abstract

**Background:**

The underlying mechanisms of alcohol use disorder (AUD) are regarded to be strongly associated with genetic factors. Although great efforts have been made to identify the association of rs4680 polymorphism in the catechol-o-methyltransferase gene and risk to AUD, the outcomes were still inconsistent. This study is aimed at exploring the association of rs4680 polymorphism and AUD by using a meta-analysis approach.

**Methods:**

Literature searching was undertaken across PubMed, Embase, Web of Science, Chinese National Knowledge Infrastructure (CNKI), and Wanfang databases. We set the search period before February 20, 2020. We used the Review Manager 5.3 (RevMan 5.3) software to estimate the effect sizes in five genetic models.

**Results:**

In total, eighteen case-control studies and two cohort studies were included in this study. The merged results of overall population indicated there was no significant association between rs4680 polymorphism and AUD: V vs. M, OR = 1.02, 95% CI 0.93-1.12, *P* = 0.70; VV vs. MM, OR = 0.99, 95% CI 0.79-1.23, *P* = 0.92; VM vs. MM, OR = 0.91, 95% CI 0.81-1.03, *P* = 0.15; VV+VM vs. MM, OR = 0.95, 95% CI 0.80-1.13, *P* = 0.65; VV vs. VM+MM, OR = 1.04, 95% CI 0.91-1.18, *P* = 0.57. Subgroup analysis by gender suggested rs4680 polymorphism was marginally associated with an elevated risk to AUD among males (VM vs. MM, OR = 0.81, 95% CI 0.67-0.98, *P* = 0.03). However, subgroup analysis by race and diagnosis did not support any significant association.

**Conclusions:**

The present study suggests that rs4680 polymorphism has no association with AUD in the overall population, but it has a weak association with AUD in males. Carriers of VM genotype in males appear to have an increased risk to AUD.

## 1. Introduction

Alcohol use disorder (AUD) is a chronic relapsing psychiatric disorder manifested by excessive alcohol consumption, which often results in physical and psychological symptoms [1]. Obsessive and compulsive use of alcohol is widely seen in various countries and ethnicities. Statistics from the World Health Organization (WHO) displayed that about 3 million people die from alcohol-related diseases every year. About 3.8% of global deaths and 4.6% of global disability-adjusted life years lost are caused by excessive alcohol consumption, thus leading to enormous economic burden and huge health problems [2]. Despite all these efforts on the study of AUD have been made in recent years, the exact etiology and pathogenesis of AUD are yet an unanswered question. Therefore, the investigation of the underlying molecular mechanisms of AUD may help us explore preventive and treatment measures.

Evidence in many studies showed that more than 50% of the risk factor could be attributed to genetic components [3, 4]. The association of Val158Met (rs4680) polymorphism in catechol-O-methyltransferase (COMT) and AUD was first investigated by a Japanese study [5]. Another study published in 2004 indicated that rs4680 polymorphism has a significant influence on enzyme activity, the enzyme activity of COMT-Met is about 40% lower than that of COMT-Val in human dorsolateral prefrontal cortex at normal physiological temperature [6], and this may influence neurotransmitters dopamine, epinephrine, and norepinephrine signaling.

COMT is a protein-coding gene located on human chromosome 22q11.2. Its classical function is to catalyze the transfer of methyl groups from S-adenosylmethionine to catecholamines. This O-methylation leads to the major degradative pathway of the catecholamine transmitters and thus regulates the metabolisms of neurotransmitters dopamine, epinephrine, and norepinephrine. Polymorphisms in COMT may alter its expression level and enzyme activity. The past several decades have seen numerous investigations about the association of rs4680 polymorphisms in COMT and the risk of AUD, but their results are inconsistent. Therefore, the present work is aimed at providing a comprehensive analysis of individual studies and elucidating the correlation of rs4680 polymorphism and AUD susceptibility.

## 2. Methods

### 2.1. Literature Search

We searched Web of Science, PubMed, Embase, Wanfang, and CNKI databases. The search string was (Polymorphism OR SNPs OR Mutant OR Variant OR “Polymorphism, Single Nucleotide”[Mesh]) AND (Alcohol Dependence OR Alcohol Addiction OR Chronic Alcoholic Intoxication OR Alcohol Use Disorder OR Alcohol Use Disorders OR Alcohol Abuse OR Alcohol misuse OR Problem drinking OR Harmful drinking OR Alcoholics OR Alcoholism) AND (Catechol-O-Methyltransferase OR Catechol O Methyltransferase OR Catechol Methyltransferase OR COMT OR Catecholamine-O-methyltransferase OR Val108/158Met OR rs4680 OR “Catechol O-Methyltransferase”[Mesh]). Language restriction was not posed. The latest search was performed on February 20, 2020.

### 2.2. Inclusion and Exclusion Criteria

Studies that met the following criteria were included: (1) case-control or cohort studies on rs4680 polymorphism and AUD; (2) studies with available data for calculating odds ratio (OR) and 95% confidence interval (95% CI). Letters, editorials, duplicate studies, and case reports were excluded. All studies were reviewed by two investigators independently. Any discrepancy was resolved by mutual consent.

### 2.3. Data Extraction and Quality Assessment

Two investigators abstracted main information from the eligible studies: author's name, publication year, nation, ethnicity, gender, design, diagnostic criteria, sample size, genotype and/or allele distribution, and results of the Hardy-Weinberg equilibrium (HWE) test [7]. The quality of eligible studies was evaluated based on the Newcastle-Ottawa Scale (NOS). The NOS contained eight items, which were categorized into three boards including selection, comparability, and exposure. The studies with five or more scores were considered to be in high quality. Discrepancies were addressed by mutual consent.

### 2.4. Statistical Analysis

ORs together with 95% CIs were calculated to appraise the association of rs4680 polymorphism and alcohol use disorder under five genetic models including allelic model (V vs. M), homozygous model (VV vs. MM), heterozygous model (VM vs. MM), dominant model (VV+VM vs. MM), and recessive model (VV vs. VM+MM). We used the *Q*-statistical test and *I*^2^ test to check the between-study heterogeneity. In the condition of *P* < 0.1, *I*^2^ > 50%, the random-effected model was selected. Otherwise, the fixed-effects model was used. Subgroup analyses by ethnicity and gender were performed. Publication biases were assessed by funnel plots.

## 3. Results

### 3.1. Literature Search

**Figure 1:**
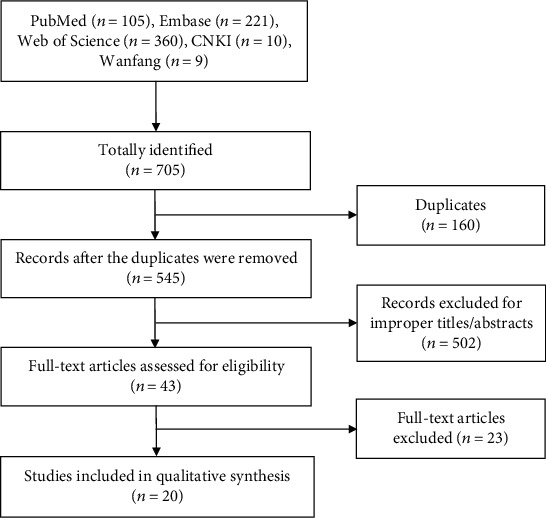
Flow diagram of literature search and screen.

In total, we identified 705 records. Of them, 180 records were erased because of duplication. After title and abstract screening, 502 irrelevant items were deleted. Of the remaining 43 publications, 23 ineligible publications were excluded based on inclusion and exclusion criteria. Ultimately, 20 articles [5, 8–26] remained for data combination. The study flow diagram is presented in Figure 1.

### 3.2. Characteristics of Eligible Studies

The main characteristics of the included studies are listed in Table 1. A total of 20 studies [5, 8–26] were included in this meta-analysis, including 18 case-control studies [5, 8, 10–14, 16–26] and 2 cohort studies [9, 15]. The studies were published between 1999 and 2017. Eighteen studies [27–32] were published in English, one [33] was in Chinese, and one [10] was in Korean. The study by Soyka et al. [23] consisted of a German cohort and a Poland cohort. Three of the studies were on alcohol abuse, and the rest of the studies were about alcohol dependence.

All the studies were in HWE with the exception of the study by Altintoprak et al. [8]. It should be pointed out that the participants in Zhang et al.'s study [26] were from different ethnicities. According to the NOS, the included studies received five or more stars (Table 2).

### 3.3. Meta-Analysis and Subgroup Analysis

**Figure 2:**
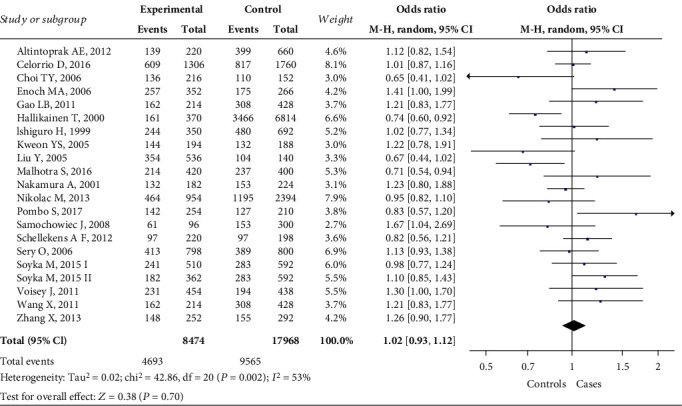
Forest plot of rs4680 polymorphism and alcohol use disorder under the allele model of inheritance in the overall population.

Overall, 20 studies with 21 independent cohorts were included in quantitative analysis. The merged data indicated no significant association between rs4680 polymorphism and AUD under five genetic models in the overall population: V vs. M, OR = 1.02, 95% CI 0.93-1.12, *P* = 0.70 (Figure 2); VV vs. MM, OR = 0.99, 95% CI 0.79-1.23, *P* = 0.92; VM vs. MM, OR = 0.91, 95% CI 0.81-1.03, *P* = 0.15; VV+VM vs. MM, OR = 0.95, 95% CI 0.80-1.13, *P* = 0.65; VV vs. VM+MM, OR = 1.04, 95% CI 0.91-1.18, *P* = 0.57.

Subgroup analysis by ethnicity did not find any significant association of rs4680 and AUD in both Asians and Caucasians under any genetic model. Furthermore, we stratified the participants by gender. For the males, a marginally significant association was observed under the heterozygous model (VM vs. MM, OR = 0.81, 95% CI 0.67-0.98, *P* = 0.03). However, no significant association was detected under other genetic models. Regarding the females, rs4680 did not appear to be associated with AUD under five genetic models. Besides, subgroup analysis based on diagnosis still did not support any association of rs4680 and AUD. The summarized outcomes are displayed in Table 3.

### 3.4. Sensitivity Analysis and Publication Bias

**Figure 3:**
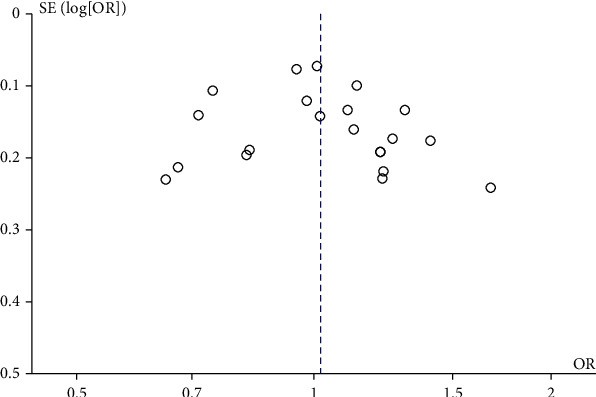
Funnel plots of rs4680 polymorphism and alcohol use disorder under the allele model of inheritance in the overall population.

After excluding the studies which were out of HWE, the recalculated effect sizes had no significant change. Therefore, it was not removed from the meta-analysis. The leave-one-out method was used to investigate the effect of an individual study on the pooled ORs and 95% CI. The results did not alter significantly through omitting any single study, indicating the stability of the outcomes. Furthermore, funnel plots did not have substantial asymmetry (Figure 3), suggesting there was no significant evidence of publication bias.

## 4. Discussion

Numerous meta-analysis studies have reported the associations of gene polymorphisms and AUD. For instance, Zhang et al.'s study [34] published in 2019 indicated that rs6296 polymorphism in the 5-HT1B gene was not associated with alcoholism. Villalba et al.'s study [35] in 2015 displayed that SLC6A4 promoter polymorphism was not associated with the risk for AUD, while Munafò et al.'s study [36] demonstrated that there was a significant but small association of the DRD2 Taq1A polymorphism with alcoholism. To our knowledge, this is the first meta-analysis study on rs4680 polymorphism in the COMT gene and AUD. We performed overall analysis and subgroup analysis by gender, ethnicity, and diagnosis. Our results suggested that rs4680 polymorphism in the COMT gene was not significantly linked to AUD in the overall population. However, a weak association was observed in males; carriers of VM genotype appeared to have an increased risk to AUD.

SNP rs4680 is a famous functional variant in the COMT gene. More than 40% of the enzymatic activity is affected by rs4680 polymorphisms. Numerous psychobiological disorders and clinical symptoms were proved to be associated with COMT gene polymorphisms. Gervasini et al.'s study [37] suggested COMT gene polymorphisms may contribute to the psychopathological symptoms of bulimia nervosa patients. Another study identified that different COMT genotypes may result in different motor behaviors [38]. A study in 2015 indicated that polymorphism in the COMT gene was associated with Parkinson's disease in a Japanese population. Apart from psychiatric disorders, COMT gene polymorphisms were also relevant to cancer risk. The relationship between functional polymorphisms in COMT and the risk of breast cancer is investigated by numerous studies all over the world. However, the results were inconsistent and contradictory. An updated meta-analysis on this issue published in 2012 suggested that rs4680 polymorphism is not associated in breast cancer susceptibility [39]. Another meta-analysis study in 2020 showed that there was no association between rs4680 polymorphism and endometrial cancer risk [40].

In the present work, we included 20 articles worldwide, of which 3 studies were from China. Most of these studies showed that there was no statistically significant association between COMT gene polymorphisms and AUD risk. After combining the results of all included studies, the pooled meta-analysis results remained the same. We also conducted the subgroup analysis divided by ethnicity and gender; all the results were turned out to be negative. Therefore, we could only come to a conclusion that there was no association between COMT gene polymorphisms and alcohol dependence risk.

COMT was first reported in 1958 [41]. COMT has multiple SNPs; rs4680 polymorphism is among them. rs4680 polymorphism could significantly affect protein abundance and enzyme activity, but it could not alter the mRNA expression levels. The different enzyme activity between alleles may be attributed to differences in protein integrity. Chen et al.'s study [6] demonstrated that the influence of rs4680 SNP on COMT activity is independent from three other SNPs in the COMT gene.

COMT mainly prevails as an S isoform in the cytoplasm. It is widely distributed in most tissues including the liver and pituitary gland, cerebellum, lungs, mammary glands, etc. [42, 43]. Guanine-to-adenine transition at codon 158 accounts for valine (Val) to methionine (Met) substitution, thus leading to the alteration of COMT enzyme activity. Val/Val, Val/Met, and Met/Met genotypes represent high, medium, and low activity, respectively, since the COMT enzyme is essential to dopamine degradation. It is possible that this functional polymorphism may contribute to the underlying molecular mechanisms of AD.

Some drawbacks of the present work should not be neglected. First, although we have gathered the most comprehensive original studies on this issue worldwide, the study number and the sample size are relatively small and thus may lead to a possibility of false negative. Second, it is clear that COMT polymorphisms have significant impact on the enzyme activity. However, we did not found any association between rs4680 polymorphism and AUD susceptibility in the overall population; the underlying mechanisms are yet an unanswered question. Third, the occurrence of AUD is thought to be determined by the combined effects of the intrinsic factor and environment factor; we only concentrated on the genetic influences. Fourth, the *P* value for the HWE test of one included study is <0.05, which means that the included participants of this study may not be representative. Last but not the least, we only searched the online databases in English and Chinese; relevant articles written in other languages were not included in this work, which may lead to selection bias.

## 5. Conclusion

The present study suggests that rs4680 polymorphism has no association with AUD in the overall population, but it has a weak association with AUD in males. Carriers of VM genotype in males appear to have an increased risk to AUD. Considering the limitations of this study, future well-designed studies with large sample sizes are encouraged.

## Figures and Tables

**Table 1:** Characteristics of included studies.

Author/year	Nation	Ethnicity	Diagnosis	Gender	Design	Diagnostic criteria	Sample size	Case					Control					HWE (*P* value)
								V	M	VV	VM	MM	V	M	VV	VM	MM	
Altintoprak AE, 2012	Turkey	Asian	AD	Both	Case-control	DSM-IV	110/330	139	81	47	45	18	399	261	137	125	68	<0.01
Celorrio D, 2016	Spain	Caucasian	AA	Both	Cohort	Altisent	648/864	609	697	NA	NA	NA	817	943	NA	NA	NA	0.63
Choi TY, 2006	Korea	Asian	AD	Male	Case-control	DSM-IV	108/76	136	80	45	46	17	110	42	38	34	4	0.30
Enoch MA, 2006	USA	Caucasian	AD	Both	Case-control	DSM-III-R	176/133	257	95	91	75	10	175	91	59	57	17	0.58
Gao LB, 2011	China	Asian	AD	NA	Case-control	DSM-IV	107/214	162	52	61	40	6	308	120	106	96	12	0.10
Hallikainen T, 2000	Finland	Caucasian	AD	Male	Case-control	DSM-III-R	185/3407	161	209	32	97	56	3466	3348	880	1706	821	0.92
Ishiguro H, 1999	Japan	Asian	AD	Both	Case-control	DSM-IV	175/346	244	106	85	74	16	480	212	166	148	32	0.91
Kweon YS, 2005	Korea	Asian	AD	Male	Case-control	DSM-IV	97/94	144	50	54	36	7	132	56	47	38	9	0.75
Liu Y, 2005	Japan	Asian	AA	Both	Cohort	NA	268/70	354	182	115	124	29	104	36	40	24	6	0.39
Malhotra S, 2016	India	Asian	AD	Male	Case-control	DSM-IV	210/200	214	206	51	112	47	237	163	64	109	27	0.07
Nakamura A, 2001	Japan	Asian	AD	Male	Case-control	DSM-III	91/112	132	50	45	42	4	153	71	49	55	8	0.16
Nikolac M, 2013	Croatia	Caucasian	AD	Both	Case-control	DSM-IV	477/1197	464	490	117	230	130	1195	1199	289	617	291	0.29
Pombo S, 2017	Portugal	Caucasian	AD	NA	Case-control	DSM-IV-TR	127/105	142	112	48	46	33	127	83	39	49	17	0.81
Samochowiec J, 2008	Poland	Caucasian	AD	Both	Case-control	ICD-10	48/150	61	35	20	21	7	153	147	42	69	39	0.33
Schellekens AF, 2012	Netherlands	Caucasian	AD	Male	Case-control	DSM-IV	110/99	97	123	26	45	39	97	101	22	53	24	0.48
Sery O, 2006	Czech	Caucasian	AD	Both	Case-control	DSM-IV	399/400	413	385	107	199	93	389	411	93	203	104	0.75
Soyka M, 2015 I	German	Caucasian	AD	Both	Case-control	DSM-IV	255/296	241	269	57	127	71	283	309	67	149	80	0.88
Soyka M, 2015 II	Poland	Caucasian	AD	Both	Case-control	DSM-IV	181/296	182	180	48	86	47	283	309	67	149	80	0.88
Voisey J, 2011	Australia	Caucasian	AD	Both	Case-control	DSM-IV	227/219	231	223	NA	NA	NA	194	244	NA	NA	NA	0.05
Wang X, 2011	China	Asian	AD	Both	Case-control	DSM-IV	107/214	162	52	61	40	6	308	120	106	96	12	0.10
Zhang X, 2013	China	Mixed	AA	Both	Case-control	DSM-IV	126/146	148	104	41	66	19	155	137	41	73	32	0.96

AD: alcohol dependence; AA: alcohol abuse; V: Val; M: Met; HWE: Hardy-Weinberg equilibrium; NA: not available.

**Table 2:** Quality assessment of included studies.

Study	Adequate definition of cases	Representativeness of cases	Selection of control subjects	Definition of control subjects	Control for important factor	Exposure assessment	Same method of ascertainment for all subjects	Nonresponse rate	Total
Altintoprak AE, 2012	1	0	0	1	2	1	1	1	7
Celorrio D, 2016	1	1	1	1	2	1	1	1	9
Enoch MA, 2006	1	1	1	1	2	1	1	1	9
Gao LB, 2011	1	0	0	1	1	1	1	1	6
Hallikainen T, 2000	1	0	0	1	1	1	1	1	6
Ishiguro H, 1999	1	0	0	1	2	1	1	1	7
Kweon YS, 2005	1	0	1	1	1	1	1	1	7
Liu Y, 2005	0	1	1	1	2	1	1	1	8
Malhotra S, 2016	1	0	0	1	1	1	1	1	6
Nakamura A, 2001	1	0	1	1	1	1	1	1	7
Nikolac M, 2013	1	0	0	1	1	1	1	1	6
Pombo S, 2017	1	0	0	1	0	1	1	1	5
Samochowiec J, 2008	1	0	1	1	2	1	1	1	8
Schellekens A F, 2012	1	1	1	1	2	1	1	1	9
Sery O, 2006	1	0	0	1	2	1	1	1	7
Soyka M, 2015	1	0	1	1	2	1	1	1	8
Voisey J, 2011	1	0	1	1	1	1	1	1	7
Wang X, 2011	1	0	0	1	2	1	1	1	7
Choi TY, 2006	1	0	0	1	2	1	1	1	7
Zhang X, 2013	1	1	1	1	0	1	1	1	7

**Table 3:** Association between rs4680 polymorphism and alcohol use disorder.

Genetic model	Association			No. of cohorts	Effect model	Heterogeneity	
	OR	95% CI	*P* value			*I* ^2^ (%)	*P* value
Overall							
V vs. M	1.02	0.93-1.12	0.70	21	R	53	<0.01
VV vs. MM	0.99	0.79-1.23	0.92	19	R	52	<0.01
VM vs. MM	0.91	0.81-1.03	0.15	19	F	26	0.14
VV+VM vs. MM	0.95	0.80-1.13	0.56	19	R	44	0.02
VV vs. VM+MM	1.04	0.91-1.18	0.57	19	R	34	0.08

Asian							
V vs. M	0.97	0.82-1.16	0.75	9	R	51	0.04
VV vs. MM	0.85	0.65-1.11	0.23	9	F	40	0.10
VM vs. MM	0.87	0.67-1.14	0.33	9	F	3	0.41
VV+VM vs. MM	0.86	0.67-1.11	0.25	9	F	27	0.20
VV vs. VM+MM	0.99	0.85-1.16	0.90	9	F	40	0.10

Caucasian							
V vs. M	1.03	0.92-1.16	0.61	11	R	59	<0.01
VV vs. MM	1.02	0.76-1.36	0.90	9	R	62	<0.01
VM vs. MM	0.91	0.74-1.12	0.37	9	R	42	0.09
VV+VM vs. MM	0.94	0.76-1.17	0.60	9	R	55	0.02
VV vs. VM+MM	1.04	0.91-1.19	0.54	9	F	39	0.11

Male							
V vs. M	0.92	0.81-1.05	0.24	11	R	45	0.05
VV vs. MM	0.87	0.60-1.24	0.43	10	R	57	0.01
VM vs. MM	0.81	0.67-0.98	0.03	10	F	30	0.17
VV+VM vs. MM	0.84	0.64-1.10	0.21	10	R	46	0.06
VV vs. VM+MM	0.92	0.78-1.08	0.30	10	F	30	0.17

Female							
V vs. M	0.98	0.78-1.23	0.84	5	R	64	0.03
VV vs. MM	0.84	0.47-1.48	0.54	4	R	56	0.08
VM vs. MM	0.85	0.66-1.09	0.21	4	F	0	0.47
VV+VM vs. MM	0.83	0.65-1.05	0.12	4	F	32	0.22
VV vs. VM+MM	0.91	0.72-1.13	0.39	4	F	49	0.12

Alcohol dependence							
V vs. M	1.03	0.92-1.14	0.63	18	R	55	<0.01
VV vs. MM	0.98	0.78-1.23	0.85	17	R	53	<0.01
VM vs. MM	0.90	0.79-1.01	0.08	17	F	27	0.14
VV+VM vs. MM	0.93	0.78-1.11	0.43	17	R	45	0.02
VV vs. VM+MM	1.06	0.93-1.20	0.39	17	F	26	0.39

Alcohol abuse							
V vs. M	0.98	0.75-1.28	0.87	3	R	61	0.07
VV vs. MM	1.05	0.38-2.91	0.92	2	R	66	0.09
VM vs. MM	1.37	0.80-2.35	0.26	2	F	0	0.56
VV+VM vs. MM	1.25	0.76-2.07	0.38	2	F	37	0.21
VV vs. VM+MM	0.84	0.39-1.80	0.65	2	R	77	0.04

OR: odds ratio; CI: confidence interval; F: fixed-effects model; R: random-effects model.

## Data Availability

All analyses of this work were based on previously published literatures and public databases.
